# Marine phytoplankton community composition data from the Belgian part of the North Sea, 1968–2010

**DOI:** 10.1038/sdata.2018.126

**Published:** 2018-07-03

**Authors:** Anja Nohe, Carolien Knockaert, Annelies Goffin, Elien Dewitte, Karien De Cauwer, Xavier Desmit, Wim Vyverman, Lennert Tyberghein, Ruth Lagring, Koen Sabbe

**Affiliations:** 1 University of Ghent, Department of Biology, Laboratory of Protistology & Aquatic Ecology, Krijgslaan 281 (S8), 9000 Gent, Belgium; 2 Flanders Marine Institute, Wandelaarkaai 7, 8400 Oostende, Belgium; 3 Belgian Marine Data Centre (BMDC), Operational Directorate Natural Environment, Royal Belgian Institute of Natural Sciences, Gulledelle 100, 1200 Brussels, Belgium

**Keywords:** Biodiversity, Water microbiology, Marine biology

## Abstract

The Belgian Phytoplankton Database (BPD) is a comprehensive data collection comprising quantitative phytoplankton cell counts from multiple research projects conducted since 1968. The collection is focused on the Belgian part of the North Sea, but also includes data from the French and the Dutch part of the North Sea. The database includes almost 300 unique sampling locations and more than 3,000 sampling events resulting in more than 86,000 phytoplankton cell count records. The dataset covers two periods: 1968 to 1978 and 1994 to 2010. The BPD can be accessed online and provides high quality phytoplankton count data. The species taxonomy is updated, and the count values are quality checked and standardized. Important metadata like sampling date, sampling location, sampling depth and methodology is provided and standardized. Additionally, associated abiotic data and biovolume values are available. The dataset allows to conduct analyses of long-term temporal and spatial trends in phytoplankton community structure in the southern part of the North Sea, including changes in phytoplankton phenology and seasonality.

## Background & Summary

Because of its location in a densely populated region with intensive economic activities, the North Sea has been seriously affected by anthropogenic activities, both historical and contemporary^1,2^. Especially in the last 50 years it has been heavily impacted by pollution (e.g. heavy metals), eutrophication (with shifts in nutrient ratios), climate change, fisheries (with concomitant changes in food webs) and other disturbances (e.g. the construction of offshore windfarms)^3–7^.

Due to its role as the main primary producer in the ocean, phytoplankton influences almost all higher trophic levels, from copepod herbivores to zooplankton carnivores, pelagic fish, seabirds and marine mammals^8^. Phytoplankton is sensitive to anthropogenic pressures and both its production and composition can change as a result of eutrophication and temperature changes, but also as a result of top-down effects of changes in higher trophic levels (e.g. through fisheries, shifts in zooplankton composition)^4,5,9^. Long-term data on phytoplankton community structure offer a unique opportunity to study the impact of various anthropogenic pressures on phytoplankton, and how phytoplankton may respond to future changes.

While in most North Sea countries such as the Netherlands, France, Germany and the United Kingdom long-term monitoring phytoplankton programs have been running for several decades^10–19^, in Belgium no such structured long-term monitoring effort exists. Nevertheless, an impressive amount of phytoplankton community structure studies have been conducted during the last decades, including the 1970’s for which data are often lacking in neighbouring countries^12,13,17–19^. These historical and recent cell count data were until now scattered in technical reports or in digital form on laboratory computers only, while some data were directly incorporated in the database of the Belgian Marine Data Centre (BMDC). As a result, these quantitative phytoplankton community structure datasets, which took a lot of resources and expert knowledge to acquire, were never published or disseminated as a whole to the wider scientific community. In addition, because the data were collected by different researchers over a long time period, the data were never quality controlled or standardized in a uniform way.

**Figure 1:**
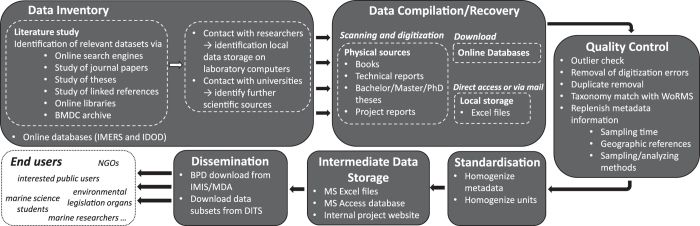
Representation of the workflow starting from the data source identification up to the final dissemination of the Belgian Phytoplankton Database (BPD).

This work is part of the 4DEMON project (www.4demon.be/), which has the aim to safeguard and centralize these valuable historical data for the future and make them available to the scientific community. To this end, we identified relevant data sources based on literature research, contacted researchers, digitised data values, assembled metadata, conducted quality control on the data and integrated the data in an extensive database for the BPNS, the Belgian Phytoplankton Database (BPD) (Fig. 1) (Data Citation 1). The BPD is available through the Integrated Marine Information System (IMIS) hosted at the VLIZ (Flanders Marine Institute).

## Methods

### Data Inventory

Possible data sources were identified based on literature research, web-based search engines and queries in online databases such as IMIS (Integrated Marine Information System - www.vliz.be/en/imis), IMERS (Integrated Marine Environmental Readings & Samples - www.vliz.be/vmdcdata/imers) and IDOD (Integrated and Dynamical Oceanographic Data management - http://www.bmdc.be). In addition, universities and researchers were contacted by mail, phone or personally. All data sources are inventoried in the Data Inventory and Tracking System (DITS - http://dits.bmdc.be) managed by BMDC. The majority was made available through IMIS via www.vliz.be/en/imis?module=ref&SpCol=809&show=search.

### Data compilation

After the identification of relevant data sources, all non-digitally available data sources (namely books, technical reports, Bachelor theses, Master theses, PhD theses and project reports) were scanned. The data values were manually transferred to a standard format in MS Excel. Data were also downloaded or extracted from databases. We directly accessed data already available in digital format on laboratory computers or received them from researchers. All compiled data were integrated in a MS Access database.

### Quality Control & standardization

#### Metadata

A significant amount of metadata was not easily accessible via the data sources themselves. An intensive effort was made to recover all relevant metadata e.g. station information or methodological approaches from associated sources such as final project reports.

#### Taxonomy

During the last decades there were many extensive nomenclatural and other taxonomic revisions of phytoplankton taxa based on progress related to advances in microscopy and molecular-phylogenetic analyses. For this reason, species names needed to be referenced prior to inclusion in the database. This was done using the taxon match option available in the World Register of Marine Species (www.marinespecies.org), a universally recognized and authoritative open-access reference system for marine species managed by VLIZ and edited by more than 240 taxonomic editors world-wide. Every species name has a unique identifier known as the AphiaID^20^. This identifier enables to link the species name to an internationally accepted standardized name and associated taxonomic information, but also redirects to the most accurate information on the species taxonomy, like accepted names and synonyms.

The taxon match was conducted in November 2017. Due to spelling mistakes present in the original reports or resulting from errors during digitisation (caused by illegible or low quality handwriting in the original paper reports), many names were not recognized automatically by the World Register of Marine Species (WoRMS), but were matched manually. Finally, less than 1% of the records could be matched neither automatically nor manually. In these cases the ‘AphiaID matched’ field stayed empty, but the records were not discarded.

A thorough clean-up of the species names and manual matching yielded a total of 99% of the taxon names being referenced with an AphiaID. Some species which were not listed in the WoRMS database were, after approval of the dedicated editor, added to this register.

#### Geographic reference

For most of the stations geographical coordinates were available or could be deduced from the synthesis reports or publications which made use of the data. Stations with unknown coordinates, but located on a map, were georeferenced in QGIS or based on standards within Marine Regions (www.marineregions.org). Stations from the same project which had slightly different names in various paper sources were compared and a unique station name was assigned. Finally, 93.8% of the records were assigned to stations with geographical coordinates.

#### Analyses and sampling methodologies

Phytoplankton was sampled using Niskin bottles, but also using other unspecified types of recipients (e.g. bottles, buckets) or a Van Dorn-sampler (Table 1). The samples were preserved with Lugol’s solution, formaline or natrium acetate. They were cooled or stored at room temperature and protected from the light. Cells were consistently counted with the Utermöhl method^21^ using the inverted microscope as optical instrument; for the large dinoflagellate *Noctiluca scintillans* sometimes a stereoscopic microscope was used. Sampling techniques, preservation steps and analytical methods are described in detail in the metadata of the BPD.

#### Additions and changes

The data were screened for random digitisation errors (mistakes made during transfer from handwritten documents to digital format). Duplicate values, resulting from data sources reporting on the same data, were removed. All zero values were removed. All units were converted to the common unit cells per litre. *Phaeocystis* cells associated in colonies are in the unit ‘10^6^ coc L^-1^‘ (= colonial cells per litre). For 249 common phytoplankton species (168 Bacillariophyceae, 76 Dinophyceae, 4 Prymnesiophyceae and 1 Cryptophyceae) biovolume calculations based on literature values and online sources are given as additional information.

## Data Records

### Historic Projects

The BPD is a compilation of data assembled from different research projects conducted since 1968. At the end of the 1960s and in the 1970s the University of Leuven joined cruises to Iceland to investigate the pelagic environment. In 1970 the Belgian government financed an integrated research project called Projet Mer/Projekt Zee (PMPZ) to assess the quality of the marine environment of the BPNS. This project was followed by national research programs called Concerted Research Actions (CRA) from 1977 until 1981. From 1990 onwards subsequent projects such as AMORE (Advanced Modelling and Research on Eutrophication) focused on *Phaeocystis* blooms in the English Channel and the Southern Bight of the North Sea with a strong focus on the BPNS^22–24^. In the years 2000, phytoplankton analyses were collected and processed in the framework of Bachelor and Master theses at Ghent University and in the framework of the EU Water Framework Directive (WFD) in order to study spatiotemporal dynamics in phytoplankton community structure in the BPNS^25–29^ (Table 2).

### Record types

Data recovery resulted in a high number of biotic values and associated abiotic parameters. In total 86,746 phytoplankton records are stored in the BPD. Quantitative units are phytoplankton densities in cells per litre (95.1% of the records) and abundance classes in cells per litre (4.4% of the records). Abundance classes reflect a range of cell densities per litre e.g. density between 1,000 and 9,999 cells per litre. 17,342 records are tagged with a living/dead (12,114/5,228) notation. ‘Dead’ refers to dead cells, e.g. diatom frustules without a cell content.

### Metadata

Each individual data record is linked to its associated metadata such as information about the data source, the sampling event, the sampling and analysis method, the project, the physical dataset origin and the phytoplankton taxonomy.

### Spatial & temporal coverage

**Figure 2:**
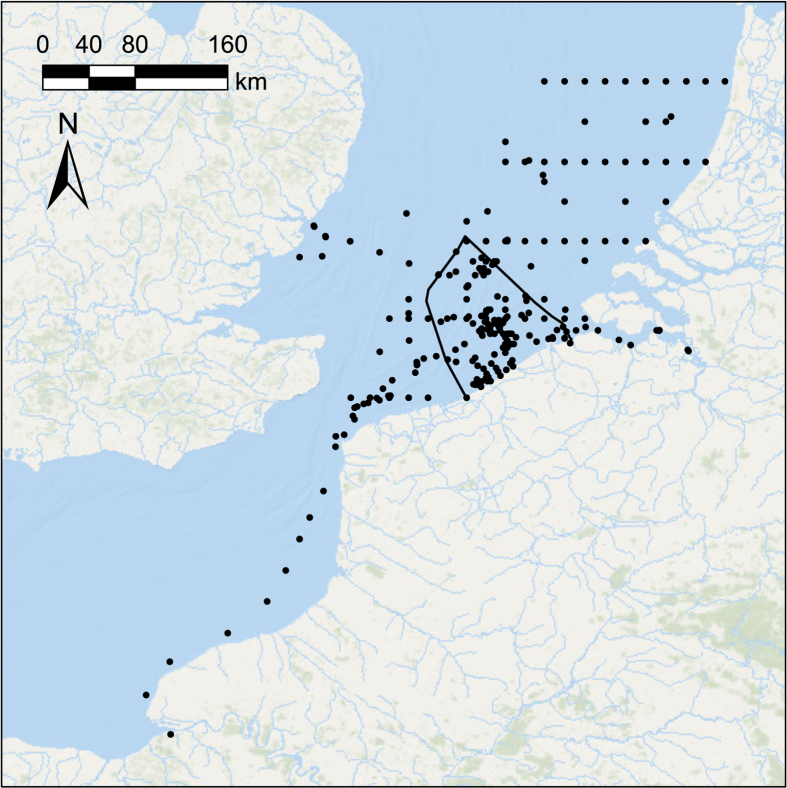
Locations of the sampling stations. Sampling stations are marked as black dots. The boundary of the Belgian part of the North Sea (BPNS) is indicated as a black line.

The database includes almost 300 individual phytoplankton sampling stations in the BPNS and adjoining areas (French, Dutch and British waters) (Fig. 2) of which 137 sampling stations are situated within the BPNS resulting in a total of 51.6% of all records deriving from samples taken in the BPNS.

**Figure 3:**
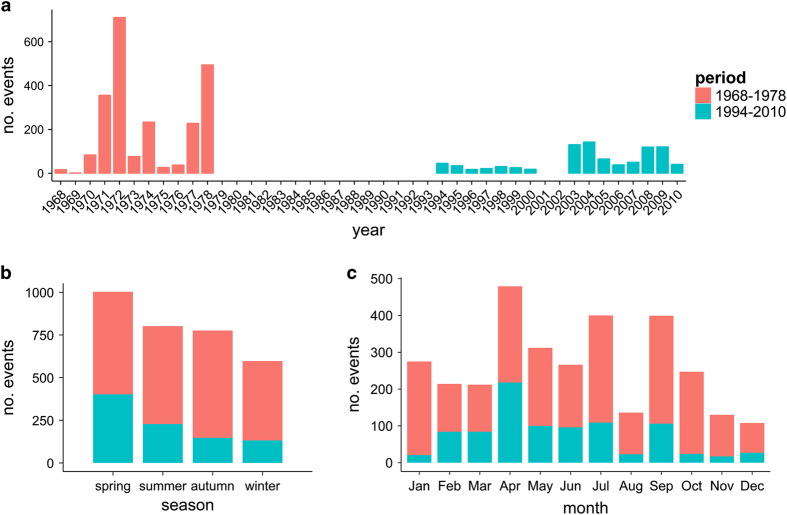
Amount of the phytoplankton sampling events. The two periods 1968-1978 (red) and 1994-2010 (blue) are distinguished. **a**: Amount of sampling events per year from 1968 to 2010. **b**: Amount of sampling events per season, **c**: Amount of sampling events per month. Winter=December-February, spring=March-May, summer=June-August, autumn=September-November.

Data are available for the years 1968 to 1978 and 1994 to 2010 (Fig. 3a). In total, 3,178 sampling events took place throughout these periods of which 1,782 took place in the BPNS. The dataset has a good seasonal coverage (Fig. 3b and Fig. 3c). Between 1968 and 1978 2,269 sampling events took place which is on average 206 events per year. These events resulted in 56,286 records, an average of 5,117 records per year and a record to event ratio of 25. Between 1994 and 2010, 909 sampling events resulted in 30,460 records, which is on average 60 sampling events and 2,031 records per year.

### Taxonomic coverage

The dataset contains 681 unique AphiaIDs of which 93% were at least identified to the genus level. The remaining 7% were identified to a higher taxonomic level or could not be matched (1%). Bacillariophyceae (diatoms) and Dinophyceae (dinoflagellates) are the two most counted phytoplankton groups representing 86.4% and 6.3% respectively of all records (Table 3).

### Associated environmental data

The associated environmental abiotic data (15,199 environmental records) measured during the same campaigns and projects in the BPNS are included, containing *i.a*. concentrations of nutrients, chlorophyll a, temperature, salinity and pH. Similar to the phytoplankton data, this is a compilation originating from various laboratories and changes in methods have occurred over the years. The data have been quality checked and referenced in time and geographically. Duplicates, outliers and zero-values have been removed. The environmental data can be either directly linked to the phytoplankton data via a common sampleID (1,230 samples, 10,332 environmental records) or via a combination of sampling date and station (726 samples, 4,867 environmental records). The latter do not share a common sampleID with the phytoplankton data e.g. because the exact sampling time during the day or the sampling depth may differ.

### Documentation and dataset dissemination

The BPD is accessible through IMIS and can be downloaded from the Marine Data Archive (MDA) (see Data Citation 1). Note that the first version of the data file (Phytoplankton_BPNS1968-2010.xlsx) does still contain zero count values, but does not yet include phytoplankton biovolume estimates or abiotic data.

## Technical Validation

The BPD contains high quality phytoplankton count data of several decades and its associated abiotic data. As the BPD is a compilation of different research projects users should be aware of the following before usage. During the last decades, different protocols were used. For example, the sample collection method, the storage of the samples and the preservation methods can differ. In addition, cell counts have been performed by several researchers. In addition to variable levels of taxonomic expertise and difference in species concepts, it is a well-known fact that taxonomic skills can improve even during the careers of single taxonomists (as a result of growing expertise, but also better analytical tools and identification guides). This personal component in microscopic taxonomic determination can never be excluded completely^30^. All data present in the BPD however were obtained in well-equipped Belgian laboratories known for their high research standards and having extensive expertise in the field of phytoplankton identification and/or taxonomy. Therefore, the phytoplankton identifications and counts are considered to be generally solid.

**Figure 4:**
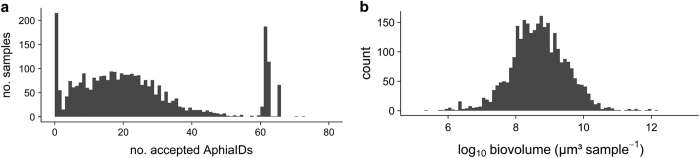
Summary statistics of the phytoplankton records. (**a**) Frequency distribution of the number of accepted AphiaIDs in the BPD, (**b**) Histogram of the logarithmically transformed biovolumes (log_10_ μm^3^ sample^−1^) per sample of the most common phytoplankton species.

Throughout the dataset diatom and dinoflagellate records are dominant (Table 3). Variation in taxon richness (number of accepted AphiaIDs) per sample in the BPD is shown in Fig. 4a. The peaks at 1 and around 60 can be attributed to specific projects. For example, the AMORE, AMOREII_VUB-ECOL and IPMS-PHAEO projects only focused on a few specific groups (such as the Bacillariophyceae (as a whole), *Phaeocystis* and *Noctiluca*), which explains the large number of samples for which only a single group is reported. The peak around 60 is mainly due to the AMORE II and III projects. In these projects, many taxa per sample get the same (low) density of 100 cells L^−1^. As zero values are absent for these samples, we suspect that these entries concern some indication of the fact that these species were under the detection limit. As we cannot be sure what these values mean, we have decided to leave them as they are in the dataset.

In addition, missing metadata (e.g. coordinates of sampling location) can limit the usability of some data records. Despite these caveats, the BPD is the only Belgian phytoplankton database which contains data going back almost five decades. It is a comprehensive and thoroughly quality checked integrated data series which includes reliable data on phytoplankton in the BPNS for marine researchers and other interest groups (Fig. 4b).

## Usage Notes

**Figure 5:**
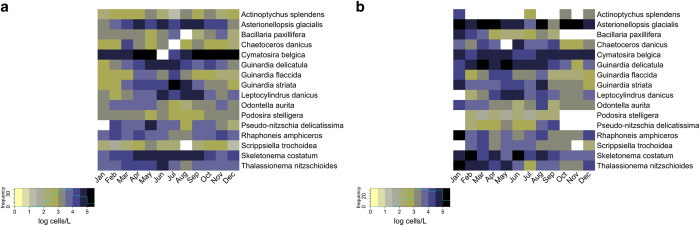
Heatmaps with colour key and histogram of the monthly logarithmic mean abundances of a selection of phytoplankton species reported in the Belgian Phytoplankton Database (BPD). Colours indicate the monthly mean logarithmic density (log_10_ cells L^−1^) of the species. **a**: Records dating from 1968 to 1978, **b**: Records dating from 1994 to 2010.

The BPD can be used to study spatio-temporal changes in phytoplankton community structure in the BPNS and adjoining areas in the period 1968-2010. Inter-annual as well as seasonal patterns can be studied (Fig. 5). Data can be analysed at the species level, but also aggregated data like e.g. total diatom or total dinoflagellate abundances can be analysed and community indices like diatom to dinoflagellate abundance ratios can be calculated. Furthermore, multivariate community analysis with e.g. ordination methods or general additive mixed modelling is an interesting field of study.

## Additional information

**How to cite this article**: Nohe, A. *et al*. Marine phytoplankton community composition data from the Belgian part of the North Sea, 1968-2010. *Sci. Data* 5:180126 doi: 10.1038/sdata.2018.126 (2018).

**Publisher’s note**: Springer Nature remains neutral with regard to jurisdictional claims in published maps and institutional affiliations.

## Supplementary Material



## Figures and Tables

**Table 1:** Sampling methodologies, preservation and analytical methods reported in the Belgian Phytoplankton Database (BPD).

Method	Sampling Instrument	Preservation	Analysis Instrument	Method Description
1	bucket, Niskin bottle	Lugol's solution; cool, dark	inverted microscope	Samples were fixed with Lugol's solution and stored cool at 4 °C in the dark. Samples were analysed 1–3 months after sampling with the Utermöhl method.
2	Nansen bottle	Lugol's solution	inverted microscope	Samples were fixed with Lugol's solution. They were analysed with the Utermöhl method. Sedimentation chambers of 50 ml and 100 ml were used (sometimes 10 ml). Magnification 320x.
3	Niskin bottle	formalin; room temperature	stereoscopic microscope	Samples were fixed with formalin. Samples were kept at room temperature and analysed with a stereoscopic microscope.
4	Niskin bottle	Lugol's solution	inverted microscope	Inverted microscope (magn. 40×10×, 60×10×, 100×10×). Some diatom species were analysed after oxiditation with an electron microscope (SEM) to detemine them on species level.
5	Niskin bottle	Lugol's solution	inverted microscope	Samples were fixed with Lugol's solution. Samples were analysed with the Utermöhl method. Some diatom species were analysed after oxiditation with an electron microscope (SEM) to detemine them on species level.
6	Niskin bottle	Lugol's solution	inverted microscope	Samples were fixed with Lugol's solution. Samples were analysed with Utermöhl method. Sedimentation time 24 h. Magnification of 20×10× or 40×10× was used. Only living cells are counted.
7	not available	not available	inverted microscope	A sample volume of 250 ml was concentrated to 5 ml by decantatation. Sample were counted with an inverted microscope at 10×20 and 10×40 magnification.
8	not available	not available	inverted microscope	Sedimentation. Normally 3 subsamples were analyzed.
9	plastic bottle, glass bottle	formol, Lugol's solution	inverted microscope	Preservation with 4% formol or Lugol's solution. Samples analysed with Utermöhl method. Samples were mixed well before transfer to sedimentation chamber. 4 hours sedimentation time. Living and dead cells were distinguished.
10	plastic bucket (surface samples), Van Dorn-sampler (depth samples)	J-JK-Na-acetaat solution	inverted microscope	Fixation with J-JK-Na-acetaat-solution. The Utermöhl method was used. 1 L of well-mixed sample was transferred to a 1-litre-measuring cylinder. 4 days sedimentation time. Supernatante was removed with a water-jet pump. Analysis with an inverted microscope, magn. 60×-1000×.
11	plastic pot	formol, Lugol's solution; dark	inverted microscope	Fixation with 2 ml 40% formol or Lugol's solution. Samples were kept in the dark. Analysis with Utermöhl method. Sedimentation of 5 ml, 10 ml, 25 ml or 50 ml of well-mixed sample. Inverted microscope with a magnification of 200x.
12	polyethylen bottle	formol	not documented	Addition of 250 ml 40% formol a sample volume of 25 L. Addition of destilled water to filtrate until a volume of 102.5 ml.
13	bucket, Niskin bottle	cool, dark	inverted microscope	Samples were fixed with Lugol's solution and stored cool at 4 °C in the dark. Samples were analysed 1–3 months after sampling with the Utermöhl method.

**Table 2:** Overview of data sources integrated in the Belgian Phytoplankton Database (BPD).

source	type	dataset source	temporal coverage	no. records	metadata link
AFVALWATEREN	book	paper	1970-1972	5,188	http://www.vliz.be/en/imis?module=ref&refid=13326
AMORE_ULB-ESA	project	www.bmdc.be	1997-2000	129	http://www.vliz.be/en/imis?module=project&proid=74
AMOREII_ULB-ESA	project	www.bmdc.be	2003-2006	12,305	http://www.vliz.be/en/imis?module=project&proid=1065
AMOREII_VUB-ECOL	project	www.bmdc.be	2003-2004	68	no link available
AMOREIII_ULB-ESA	project	www.bmdc.be	2007-2009	11,663	http://www.vliz.be/en/imis?module=project&proid=2084
Iceland Cruises	book	paper	1970-1971	1,095	http://www.vliz.be/en/imis?module=ref&refid=24711
IPMS-PHAEO_ULB-ESA	project	www.bmdc.be	1995-1996	68	http://www.mumm.ac.be/datacentre/Catalogues/datasets.php?proj=IPMS-PHAEO
MSc Thesis C. Vanlangedonck	MSc Thesis	paper	1976-1977	2,190	http://www.vliz.be/en/imis?module=ref&refid=216487
MSc Thesis E. de Block	MSc Thesis	paper	1977-1978	17,291	http://www.vliz.be/en/imis?module=ref&refid=216445
MSc Thesis M. Franck	MSc Thesis	Excel file	2003	791	http://www.vliz.be/en/imis?module=ref&refid=67322
MSc Thesis K. Töpke	integrated dataset	Excel file	2004-2006	1,399	http://www.vliz.be/en/imis?module=ref&refid=200644
PAE phytoplankton species dataset	integrated dataset	Excel file	2004	57	no link available
monitoring KRW	integrated dataset	Excel file	2007-2008	1,190	http://www.vliz.be/en/imis?module=ref&refid=289406
monitoring KRW	integrated dataset	Excel file	2009-2010	1,783	http://www.vliz.be/en/imis?module=ref&refid=203122
PhD Thesis A. M'harzi	PhD Thesis	Excel file	1994	440	http://www.vliz.be/en/imis?module=ref&refid=32158
PhD Thesis J. Smeets	PhD Thesis	paper	1974-1976	502	http://www.vliz.be/en/imis?module=dataset&dasid=4862
PhD Thesis M. Rabijns	PhD Thesis	paper	1971-1973	12,346	http://www.vliz.be/en/imis?module=ref&refid=226081
PhD Thesis R. Clarysse	PhD Thesis	paper	1968-1970	716	http://www.vliz.be/en/imis?module=ref&refid=69474
Project Sea Report	technical report	paper	1971	363	http://www.vliz.be/en/imis?module=ref&refid=240591
Project Sea Report	technical report	paper	1971	463	http://www.vliz.be/en/imis?module=ref&refid=240604
Project Sea Report	technical report	paper	1971	48	http://www.vliz.be/en/imis?module=ref&refid=240605
Project Sea Report	technical report	paper	1972	7,703	http://www.vliz.be/en/imis?module=ref&refid=240607
Project Sea Report	technical report	paper	1973-1974	1,663	http://www.vliz.be/en/imis?module=ref&refid=240648
Project Sea Report	technical report	paper	1974	1,560	http://www.vliz.be/en/imis?module=ref&refid=205357
Project Sea Report	technical report	paper	1974	5,158	http://www.vliz.be/en/imis?module=ref&refid=205359
TROPHOS_UGent-MARBIO	project	www.bmdc.be	2003	567	http://www.vliz.be/en/imis?module=project&proid=1074
Additional information on the dataset source, the temporal coverage, the number of records and a link to the metadata is provided.					

**Table 3:** Taxonomic phytoplankton classes present in the Belgian Phytoplankton Database (BPD).

class	no. records	% of records
Bacillariophyceae	74,955	86.41
Dinophyceae	5,482	6.32
Prymnesiophyceae	1,046	1.21
Chlorophyceae	831	0.96
Trebouxiophyceae	431	0.50
Chrysophyceae	426	0.49
Euglenoidea	388	0.45
Dictyochophyceae	224	0.26
Cryptophyceae	147	0.17
Cyanophyceae	134	0.15
Ulvophyceae	91	0.10
Prasinophyceae	54	0.06
Cyanobacteria incertae sedis	31	0.04
Conjugatophyceae	15	0.02
Chlorodendrophyceae	9	0.01
The total number of records and relative amount of records are reported.		
